# Genomic Prediction and Genome-Wide Association Study for Boar Taint Compounds

**DOI:** 10.3390/ani13152450

**Published:** 2023-07-28

**Authors:** Sara Faggion, Elena Boschi, Renata Veroneze, Paolo Carnier, Valentina Bonfatti

**Affiliations:** 1 Department of Comparative Biomedicine and Food Science, University of Padova, Viale dell’Università 16, 35020 Padova, Italy; elena.boschi@phd.unipd.it (E.B.); paolo.carnier@unipd.it (P.C.); valentina.bonfatti@unipd.it (V.B.); 2 Department of Animal Science, Universidade Federal de Viçosa, Viçosa 36570-999, Brazil; renata.veroneze@ufv.br

**Keywords:** indole, skatole, androstenone, GWAS, genomic prediction, GBLUP, candidate genes

## Abstract

**Simple Summary:**

Some of the compounds involved in sexual steroids’ metabolic pathways (i.e., androstenone, indole, and skatole) might accumulate in the adipose tissue of intact male pigs after sexual maturity, resulting in boar taint (BT). With a perspective future ban on surgical castration, in pig population where early slaughtering is not a viable option, the exploitation of genomic selection procedures might prevent this off-odor and off-flavor. The accuracy provided by the use of genomic information was equal or higher than the one obtained using pedigree information. This indicates that genomic selection could be beneficial for the traits investigated as it minimizes the need to collect individual measures of BT compounds. Several genomic regions, each with a small effect on BT compound concentrations, were identified. Genes previously associated with BT, reproduction traits, and fat metabolism are located in those genomic regions. Detection of candidate genes related to fat metabolism might be explained by the relationship between sexual steroid levels and fat deposition and be ascribed to the pig line investigated, selected for ham quality and not for lean growth.

**Abstract:**

With a perspective future ban on surgical castration in Europe, selecting pigs with reduced ability to accumulate boar taint (BT) compounds (androstenone, indole, skatole) in their tissues seems a promising strategy. BT compound concentrations were quantified in the adipose tissue of 1075 boars genotyped at 29,844 SNPs. Traditional and SNP-based breeding values were estimated using pedigree-based BLUP (PBLUP) and genomic BLUP (GBLUP), respectively. Heritabilities for BT compounds were moderate (0.30–0.52). The accuracies of GBLUP and PBLUP were significantly different for androstenone (0.58 and 0.36, respectively), but comparable for indole and skatole (~0.43 and ~0.47, respectively). Several SNP windows, each explaining a small percentage of the variance of BT compound concentrations, were identified in a genome-wide association study (GWAS). A total of 18 candidate genes previously associated with BT (MX1), reproduction traits (TCF21, NME5, PTGFR, KCNQ1, UMODL1), and fat metabolism (CTSD, SYT8, TNNI2, CD81, EGR1, GIPC2, MIGA1, NEGR1, CCSER1, MTMR2, LPL, ERFE) were identified in the post-GWAS analysis. The large number of genes related to fat metabolism might be explained by the relationship between sexual steroid levels and fat deposition and be partially ascribed to the pig line investigated, which is selected for ham quality and not for lean growth.

## 1. Introduction

Boar taint (BT) is a “urine/faecal-like”, unpleasant flavor and odor released during cooking or heating of pork meat, and it occurs in male pigs after puberty due to the accumulation in the adipose tissue of lipophilic components such as androstenone, skatole, and indole [1]. The accumulation of those compounds depends on a number of factors, including diet, age, sexual activity, and genetics [2,3,4,5,6,7]. Androstenone (5α-androst-16-en-3-one) is a steroid hormone produced by the testes, whereas skatole (3-methylindole) and indole (2,3-benzopyrrole) are produced by the bacterial breakdown of the amino acid tryptophane in the hind-gut [2,3,4,6].

Surgical castration of male piglets is normally performed within the first seven days of life of the animal and is largely used to prevent BT [8]. Also, castration avoids undesirable aggressive behaviors, generally observed in the entire male pigs, and meat and fat quality are better in castrates for dry-cured productions [9,10]. However, animal welfare concerns have led to the proposal of banning surgical castration in EU, driving farmers to grow intact males. Unfavorable consequences of surgical castration are also represented by (i) a marked reduction in animal feed efficiency, (ii) a lower lean meat content, and (iii) a higher content of saturated fatty acids, which, on one hand, makes castrates’ fat firmer and reduces the process of rancidity during the maturation of dry cured products, but, on the other hand, reduces the nutritional value of fat [9,11,12].

Slaughtering intact male pigs before sexual maturity can prevent BT, but it is not a viable option for pigs intended for protected designation of origin (PDO) dry-cured ham production, since the minimum age at slaughter required by product specifications to meet quality standards is after sexual maturity [13,14].

Selective breeding might provide an effective and long-term approach to control BT, supporting the transition towards the gradual elimination of surgical castration in the pig production industry. According to Ducro-Steverink [15], selective breeding could decrease BT incidence from 30% to 10% in less than 5 years. Significant genetic variation has been reported for androstenone, skatole, and indole, between and within pig lines. Heritability estimates are moderate to high, ranging from 0.46 to 0.72 for androstenone, from 0.26 to 0.50 for skatole, and from 0.29 to 0.35 for indole [7,16,17,18,19,20,21]. Positive genetic correlations between the compounds responsible for BT suggest that selection for the reduction in one BT compound might be beneficial for the reduction in the others. Genetic correlations range from 0.19 to 0.61 between skatole and androstenone [18,22,23,24,25], from 0.19 to 0.45 between indole and androstenone, and from 0.51 to 0.62 between skatole and indole [18,22].

According to Lervik et al. [26], estimated breeding values (EBV) for androstenone for different ages were reported to be positively correlated in Duroc boars aged 1–27 weeks. In addition, androstenone is correlated with testosterone. These relationships indicate that EBV may be used as early indicators of the BT levels in adult pigs and that breeding to reduce androstenone levels on the basis of EBV could have a significant impact on phenotype [26].

Routine BT compound phenotyping, required by traditional pedigree-based selection, is challenging and expensive, particularly when the number of selection candidates is large. In this context, the implementation of genomic selection procedures is greatly beneficial [27]. In some countries, BT has been successfully reduced through the use of genomic selection, and different breeding companies have produced “low-taint” breeding animals [20].

Genome-wide association studies (GWAS) revealed different quantitative trait loci (QTL) and candidate genes related to androstenone, skatole, and indole. QTL affecting BT compounds have been found on most *Sus scrofa* chromosomes (1, 2, 3, 4, 5, 6, 7, 9, 10, 12, 13, 14, and 15, as reported in Duarte et al. [7], and Botelho et al. [28]); this highlights the polygenic nature of BT, suggesting that genomic selection to decrease BT is the method of choice [29]. Detecting variants that significantly affect a given trait might improve genomic prediction accuracy of BT compounds [30], whereas the examination of post-GWAS data helps to identify candidate genes more effectively. When the GWAS detect multiple QTLs, gene network and pathway analyses contribute to elucidate the genetic regulation at the base of the biological processes underlying the traits and support the interpretation of GWAS results [31,32].

Several candidate genes, resulting from differential gene expression studies, were identified as encoding receptors and enzymes involved in the metabolism of androstenone, skatole, and indole in the liver and testis tissues [7]. Moreover, when comparing breeds and individuals with high and low levels of BT, discrepancies in the set of differentially expressed genes for androstenone, skatole, and indole levels were revealed [33]. Numerous studies showed that many of the differentially expressed genes in animals with different levels of BT compounds produced (1) phase I enzymes, such as the cytochrome P450 (Cyt-P450) family and their isoforms, (2) phase II enzymes, such as the sulfotransferases for steroid conjugation, and (3) enzymes in the testes that are responsible for the production of androstenone substrates and steroids [34,35,36,37].

So far, studies have focused on pig lines selected for lean growth, but since there is a well-known relationship between steroid hormone levels and adipogenesis [38], it is of interest to investigate associations between BT and genomic regions in pig populations where the breeding goals are addressed to enhanced ham quality and includes, with positive weights, traits related to adipogenesis.

Hence, the aims of the study were: (1) to evaluate the accuracy of traditional pedigree-based and genomic-enabled methods in the prediction of BT compound concentrations in a purebred sire line selected for carcass and ham quality traits; (2) to identify QTL regions and candidate genes associated with BT compound concentrations, through a single-trait GWAS approach.

## 2. Materials and Methods

### 2.1. Animals and Sample Collection

The study was conducted on 1075 intact young boars of the C21 Goland sire line (Gorzagri, Fonzaso, Italy), selected to enhance carcass and ham quality traits and widely used in Italy to generate commercial heavy pigs intended for PDO dry-cured ham production. For BT compounds quantification, a subcutaneous adipose tissue sample of approximately 0.5 g was collected from the neck area of each young boar (subjected to local anesthesia) using a biopsy device (SUISAG, Sempach, Switzerland [39]); samples were kept at −80 °C until the laboratory analysis. Samples were collected between January 2020 and June 2022. The average animal age and body weight at biopsy sampling were 209 ± 12 d and 153.48 ± 15.46 kg, respectively. Boar taint compound concentrations in adipose tissue is highly representative of the concentration of those compounds in plasma. Several studies have underlined how androstenone and skatole are easily transferred from plasma to the adipose tissue, such that if the concentration of those compounds in peripheral plasma increases, a high accumulation of androstenone and skatole in the adipose tissue is expected [40,41,42].

### 2.2. Boar Taint Compound Quantification

Reversed-phase HPLC with fluorescence detection was used to quantify BT compound concentrations. Sample preparation was performed following the method by Rostellato et al. [18]. Two different chromatographic separations were performed as described by Boschi et al. [43]: (1) an isocratic separation for skatole (SKA) and indole (IND) and (2) a gradient separation for androstenone (AND). The total time of the isocratic separation for SKA and IND quantification was 5 min. For AND quantification, the sample was injected after sample derivatization following the method described by Rostellato et al. [18]. The total time of the chromatographic separation was 11 min. As reported by Boschi et al. [43], the detection limit of the analysis was 0.08 μg/g, 1.21 ng/g, and 1.34 ng/g for androstenone, skatole, and indole, respectively. The residual coefficient of variation (CV) of compound concentration across days was <5.2%, whereas the residual CV of compound concentration within day of analysis was <2.6%.

The frequency distribution of BT compound concentrations, measured as ng/g for indolic compounds and as µg/g for androstenone, were skewed and then normalized through log-transformation of the original data.

### 2.3. Genotyping

A saliva sample was collected from each of the 1075 pigs and sent to GeneSeek Inc. laboratory (Lincoln, NE, USA) for DNA extraction and genotyping. Animals were genotyped using a high-density SNP chip (GGP Porcine 50K). All the animals were successfully genotyped. SNP markers with a minor allele frequency (MAF) lower than 1% and call rate lower than 95% were discarded. Missing genotypes were then imputed using the FImpute software [44] exploiting genotype data and pedigree information available for the C21 line. The final number of available SNP genotypes per animal was 29,844.

### 2.4. Genomic Predictions

Breeding values (EBV) for BT compound concentrations were predicted with pedigree-based BLUP (PBLUP) and genomic BLUP (GBLUP) methods. Variance components, heritability and pedigree-based EBVs were estimated using the Average Information Restricted Maximum Likelihood method implemented in the BLUPf90+ suite of programs [45] with the following univariate mixed linear model:

**y** = **1**µ + **Xb** + **Wa** + **e** (1)

where **y** is the vector of phenotypes (i.e., the log-transformed concentration of one BT compound in adipose tissue); µ is the average; **1** is an all-ones vector; **b** is the random effect of day of BT compound analysis (91 levels); **X** is an incidence matrix relating **y** to **b**; a is a vector of random animal additive genetic effects assumed to be N(0, A$\sigma_{a}^{2}$), where N(·) indicates a Gaussian probability density function, A is the numerator relationship matrix between animals (from pedigree data) and $\sigma_{a}^{2}$ is the additive genetic variance; **W** is an incidence matrix relating **y** to **a**; and **e** is a vector of random residuals assumed to be N(0, I$\sigma_{e}^{2}$), where I is an identity matrix of appropriate order and $\sigma_{e}^{2}$ is the residual variance. The same model was used to estimate the genomic-based EBVs (GEBVs) by GBLUP replacing the A relationship matrix with the genomic relationship matrix (G matrix) [46]. The day of BT compound analysis has been included in the model because it allowed to take into account the small variations in sample derivatization due to different reagent batches, handling and preparation.

Accuracy of PBLUP and GBLUP models was assessed in a 5-fold cross-validation. Firstly, phenotypes (log-transformed AND, IND, and SKA concentration) were adjusted for the random effect of day of BT compound analysis (**y_adj_**) using PREDICTF90 from the BLUPf90+ suite of programs [45] using model (1). The set of data was then randomly split into five equally-sized subsets. At each round, 4 out of the 5 subsets were used as a training set, masking the phenotypes of the subset that was used as a validation set, and EBVs and GEBVs were predicted for the individuals with masked phenotype. This procedure was repeated until each of the five subsets were used as a validation set. The accuracy ($R_{EBV,\;BV}$) of PBLUP and GBLUP predictions was estimated as follows:

(2) $$R_{EBV,\;BV}=\frac{r_{EBV,\;y_{adj}}}{\sqrt{h^{2}}}$$

where $r_{EBV,\;y_{adj}}$ is the Pearson correlation between the predicted EBVs and the adjusted phenotypes (**y_adj_**) and $h^{2}$ is the trait heritability. Prediction bias was assessed as the regression coefficient (slope, β) of the EBVs on the adjusted phenotypes. The Hotelling–Williams test [47] was used to test the difference in accuracy between PBLUP and GBLUP predictions.

### 2.5. Genome-Wide Association Studies for Boar Taint Compounds

A genome-wide association study (GWAS) was performed to test the association between the phenotype of each BT compound and SNP genotypes. The analyses were performed following a GBLUP GWAS procedure [48,49] using the BLUPf90+ suite of programs [45]. First, GEBV were predicted using BLUPf90+ using the variance components estimated with the pedigree-based BLUP. The analysis was performed according to the single-trait animal model (1), but the vector of random animal additive genetic effects a was assumed to be distributed as N(0, G$\sigma_{a}^{2}$), with N(·) indicating a normal probability density function, G being the genomic relationship matrix, and $\sigma_{a}^{2}$ being the additive genetic variance.

**G** was obtained as in VanRaden [46]:

(3) $$G=\frac{ZZ\prime }{2\sum_{i=1}^{m}p_{i}(1-p_{i})}$$

where **Z** is a matrix of gene content (0, 1, 2) adjusted for allele frequencies; ***m*** is the number of SNPs, and ***p_i_*** is the minor allele frequency of ***i***-th SNP.

The allele substitution effect of each SNP was then derived from GEBV using postGSf90 [50]:

(4) $$\hat{g}=\frac{1}{2\sum_{i=1}^{m}p_{i}(1-p_{i})}\;Z^{\prime }G^{-1}\hat{u}$$

where $\hat{g}$ is the vector of SNP effect and $\hat{u}$ is the vector of GEBV. *p*-values were obtained as described in Aguilar et al. [51]:

(5) $$p-value_{i}=2\;(1-\Phi \left(\left|\frac{{\hat{g}}_{i}}{sd\left({\hat{g}}_{i}\right)}\right|\right))$$

where Φ is the cumulative standard normal function and individual prediction error variances of SNP effect estimates were obtained as in Gualdrón-Duarte et al. [48]. In order to address the issue of multiple testing, the genome-wide significance threshold was adjusted at *p*-value = 0.05/m, thus applying a stringent Bonferroni correction.

Explained percentage of genetic variance was calculated using subsequent windows of adjacent SNPs within a distance of 0.4 Mb, which is the average haplotype block size in commercial pig lines [52]. Manhattan plots of GWAS results and plots of the variance explained by the SNP windows were obtained with the R package qqman [53].

### 2.6. Identification of QTLs and Candidate Genes

For each BT compound, the 0.4 Mb windows that explained 0.5% or more of the total genetic variance were selected were considered as putative QTL and selected for candidate genes search. The threshold of 0.5% was chosen based on the literature [54,55] and on the expected contribution of SNP windows [56]. Assuming that the totality of the windows explained 100% of the genetic variance and assuming an equal contribution of all windows, the expected proportion of genetic variance explained by each window would be equal to 100/N, where N is the total number of windows obtained for each trait. In total, 4040, 4037, and 4050 windows were obtained for AND, IND, and SKA, respectively; hence, the expected proportion of genetic variance explained by each window, given the assumptions stated above, would be equal to 0.025% for each trait. The threshold of 0.5% used in the present study is equal to 20 times the expected variance explained (0.025% × 20 = 0.5%).

The Ensembl VEP (variant effect predictor) tool (https://www.ensembl.org/info/docs/tools/vep/index.html; accessed on 16 September 2022) was used to determine the effect of SNPs on genes, transcripts, protein sequence and regulatory regions. Genes located within 200 kb upstream and downstream of the lead SNP were considered putative candidate genes associated with the trait. The candidate genes nearby the genome-wide significant SNPs were identified by Ensembl database (https://www.ensembl.org/; accessed on 16 September 2022) using the gene annotation information of *Sus scrofa* reference genome (Sscrofa11.1), which is publicly available at NCBI. The genomic locations were downloaded from PigQTL database (https://www.animalgenome.org/pig/; accessed on 20 September 2022) and genes within the candidate region were considered as candidate genes.

## 3. Results and Discussion

### 3.1. Descriptive Statistics for Androstenone, Indole and Skatole Concentrations

For each BT compound, the number of animals with phenotypic data, and the sample mean and standard deviation before and after log-transformation are reported in Table 1. The threshold of the compound concentrations above which carcasses are generally defined as tainted are 1.0 μg/g for AND [57,58] and 0.20–0.25 μg/g for SKA [9]; in our study, only 58.25% of the data were below the androstenone threshold, whereas 96.25% of the data were below the skatole threshold. Narrow sense heritabilities (Table 1) for BT compounds estimated from pedigree-based relationships on our experimental population were moderate (h^2^ ± SE): 0.30 ± 0.08 for AND, 0.43 ± 0.09 for IND, 0.51 ± 0.09 for SKA. The estimates indicates that moderate to high genetic gain could be obtained through selection against boar taint compounds in the evaluated population. These estimates are comparable to those reported in the literature: in Landrace, Large White, Duroc, and Pietrain breeds, estimates of the genetic parameters ranged between 0.39 and 0.73 for AND and between 0.32 and 0.69 for SKA [20]; in the pig line used the current study, heritability estimates were 0.39 for AND and 0.60 for SKA [18]. Even though estimates for IND are scarce in the literature, overall, they are comparable (0.33 [17]; 0.39 [18]) or slightly higher (0.55 [39]) than the one obtained in the current study.

### 3.2. Genomic Predictions

The Pearson correlations between predicted EBV and adjusted phenotypes ($r_{EBV,\;y_{adj}})$ ranged between 0.20 (AND) and 0.33 (SKA) for pedigree-based BLUP and between 0.27 (IND) and 0.35 (SKA) for genomic-based BLUP (Table 2). The slope of the regression of y_adj_ on EBV (β), ranged between 0.89 (AND) and 1.00 (SKA), whereas GEBV tended to be inflated (β < 0.89). The accuracies ($R_{EBV,\;BV}$) ranged between 0.36 (AND) and 0.45 (IND) for pedigree-based BLUP and between 0.42 (IND) and 0.58 (AND) for genomic-based BLUP (Table 2).

For AND, GBLUP performed significantly better than PBLUP, whereas for IND and SKA, the accuracies of PBLUP and GBLUP were not significantly different. The accuracies obtained from GBLUP for AND and SKA are in agreement with literature estimates. De Campos et al. [19] reported accuracies, measured as the correlation between the observed and the predicted phenotype divided by the square root of trait heritability, equal to 0.63 and 0.57 for AND and SKA, respectively, using a Ridge Regression BLUP method. Lukić et al. [59] and Botelho et al. [60] estimated the genomic breeding values for AND and SKA through a GBLUP approach with correlations between the predicted breeding values and the phenotypes of 0.27–0.35 and 0.21–0.49 for AND and SKA, respectively. In that study, the accuracies adjusted for the square root of heritability (0.56 and 0.76 for AND and SKA, respectively) were much higher than those obtained in this study.

In the literature, only Botelho et al. [60] have reported results of genomic prediction for IND: for a dataset consisting of records from a single-line pig population, prediction accuracy computed as the Pearson’s correlation between GEBV and phenotypes adjusted for fixed effects ranged between 0.24 and 0.26, whereas for a multi-line pig population, correlations were slightly lower (0.21–0.26). Those correlations are similar to that obtained in the present study.

### 3.3. Single-Trait Genome-Wide Association Study for Boar Taint Compounds

**Figure 1:**
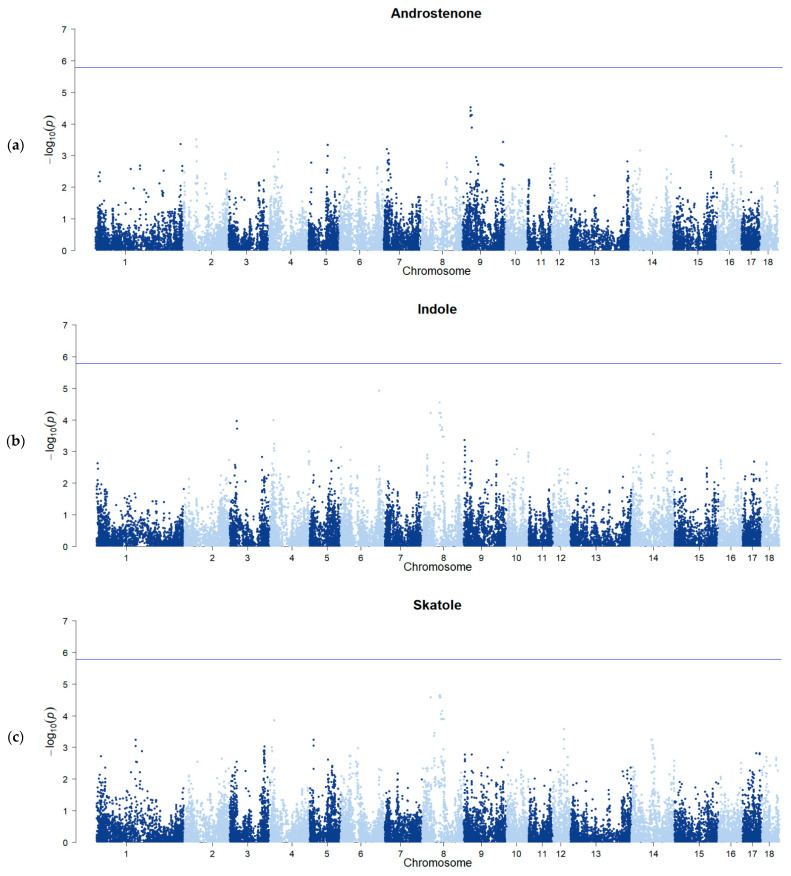
Genome-wide association plots for androstenone (**a**), indole (**b**), and skatole (**c**) concentrations. The horizontal line identifies the genome-wide significance threshold (−log_10_ (0.05/m), with m as the total number of markers).

The genome-wide significance threshold after Bonferroni correction and after −log_10_ transformation was estimated to be 5.78. The genome-wide association study failed to identify any genome-wide QTL associated to AND, IND, or SKA concentrations (Figure 1). In previous studies performed on single-line pig populations, several markers associated with variation in BT compound concentrations were detected: 33 genomic regions associated with the variation in at least one of the BT components were reported by Große-Brinkhaus et al. [29]; 14 regions for AND and 10 for SKA, in Landrace, and 14 regions for AND and 4 for SKA, in Duroc, were detected by Grindflek et al. [22].

The ability of a GWAS to identify genomic regions significantly associated with a trait variation depends on the power of the experiment, which is related to sample size and phenotype variability. The number of samples included in our study is considered sufficient for a GWAS, but might not be large enough to ensure the identification of DNA regions with a small effect on the trait. In addition, phenotype variation in our dataset was remarkably lower than that reported in other studies [22,29]. Both these aspects might have impacted our ability to detect significant associations.

When hypothesis tests are performed using a high number of SNPs, determining the multiple testing correction threshold in GWAS might be challenging; this is typically performed using the Bonferroni correction. Bonferroni correction is a highly conservative method, particularly when datasets consist of a high number of SNPs and the independence assumption does not hold, which results in a high penalty for GWAS [61]. To test the associations between BT compounds and SNP markers, different studies [28,60] have proposed an approach based on the percentage of variance explained to select markers potentially associated to BT compounds. The threshold of 0.5% was chosen based on the literature [54,55] and on the expected contribution of SNP windows [56]. Assuming an equal contribution of all windows, the expected proportion of genetic variance explained by each window would be equal to 0.025% for all the traits (100/N, where N is the total number of windows; we obtained 4040, 4037, and 4050 windows for AND, IND, and SKA, respectively). The threshold of 0.5% used in the present study is equal to 20 times the expected variance explained (0.025% × 20 = 0.5%).

**Figure 2:**
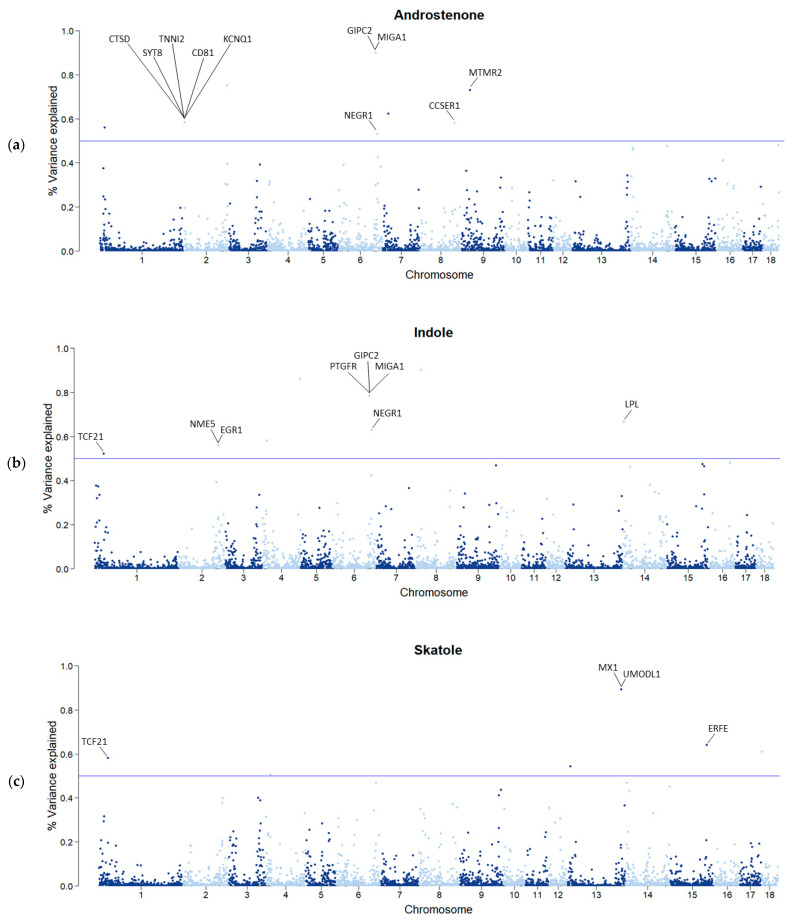
Percentage of genetic variance explained by SNP windows for androstenone (**a**), indole (**b**), and skatole (**c**) concentrations. Each dot represents one SNP window of 0.4 Mb. The horizontal line identifies the threshold of 0.5% used to select SNPs for candidate gene search.

Following this strategy, we have detected signals from several chromosome regions, most of them consistent with the literature, as well as novel chromosome regions. The percentage of variance explained by each SNP windows for AND, IND, and SKA is depicted in Figure 2. For all BT compounds, multiple genomic windows were detected, each window explaining a small amount of the trait additive genetic variance, which supports the hypothesis of polygenic nature of these traits [28,62,63,64]. In particular, 18 windows explaining more than 0.5% of the genetic variance were detected.

In a previous study performed on a single-line pig population, 37 SNPs explaining 13.7% of the variance for AND were reported [65]. Studies focused on pigs from more than one sire line led to detection of a lower number of SNP windows (16), explaining in total a small amount of genetic variance (2.90%, 2.15%, and 4.47% for AND, SKA, and IND, respectively [28]). Several authors hypothesized that variations in population stratification, genetic backgrounds, linkage disequilibrium, population size, and other factors could explain differences across studies [50,55,66]. Moreover, variation in BT varies across breeds, probably due to different selection goals for each breed. Also, a different expression of candidate genes linked to AND, SKA, and IND has been reported for individuals characterized by different levels of BT compounds or belonging to various breeds [33].

### 3.4. Identification of QTLs

For AND, QTL regions exceeding the 0.5% threshold of explained variance were identified on *Sus scrofa* chromosome 1, 2, 6, 7, 8, and 9 (Figure 2a, Table 3). Windows explained between 0.53 and 0.90% of the variance; the most relevant window was located on SSC6. Together, the eight windows identified for AND explained 5.25% of the genetic variance of the trait. Several studies have reported QTL regions associated with AND on SSC1 [28,65], 2 [28,63], 6 [22,28,62,63,65,67,68], 7 [62,63,69], and 9 [62,63], whereas the one on SSC8 has never been reported before.

For IND, windows located on SSC1, 2, 4, 6, 8, and 14 explained between 0.52 and 0.90% of the variance, with the most relevant windows located on SSC4 and 8 (Figure 2b, Table 3). The eight windows identified explained 5.50% of the genetic variance of IND. Besides the QTL regions that have been previously detected as associated with IND or pork odor (SSC2 and 6 [28,70]; SSC14 [63]), we detected additional QTLs on SSC1 and 8.

Six windows explaining 3.76% of the variance of SKA were identified on SSC1, 4, 13, 15, and 18 (Figure 2c, Table 3). Each window explained between 0.50% (on SSC4) and 0.89% (SSC13) of the variance. For SKA, one chromosome reported in the present study (SSC4) has been previously associated with the intensity of smell and taste of pork [67], whereas the QTL region in SSC13 has been associated with SKA [28]. In the current study, additional QTLs associated with SKA have been detected on SSC1, 15, and 18.

One window, common to AND and IND, located on SSC6 explained 0.53% and 0.63% of the genetic variance of the two traits, respectively. Two windows on SSC1 and SSC4 were common to IND and SKA; those windows explained 0.52% and 0.58% (SSC1) and 0.58% and 0.50% (SSC4) of the genetic variance, for IND and SKA, respectively.

### 3.5. Candidate Genes

Considering the genomic regions associated with either AND, IND, or SKA (Table 4), we identified 18 candidate genes encoding receptors and enzymes associated with BT, reproduction traits, and fat metabolism. Only one of the genes identified (MX1, *Myxovirus resistance 1*, involved in the resistance to influenza virus in pigs [71]) has been previously reported in the literature in association with BT, in particular, with the concentration of AND [37]. In the current study, the gene explained 0.89% of the variance of SKA.

The large majority of the GWAS performed on BT [22,28,29,65,72,73] identified associations with the genes encoding Cytochrome P450 family enzymes (CYP) that regulate steroid metabolic processes and are known to play a key role in the metabolism of BT compounds in the liver [5]. Other enzymes with a well-known role in BT compound metabolism (e.g., SULT [5]) have been identified by GWAS by Duijvesteijn et al. [65] and Gregersen et al. [72]. Only a few studies investigated the variance explained by SNP windows [28,29]. However, estimates of the variance explained obtained with different methods are not comparable. In the study by Botelho et al. [28], who estimated the variance explained by SNP windows using the same approach as the one used in the current study, the variance explained by the CYP candidate gene CYP27B1 was 0.56% for IND, and 0.01% for AND and SKA. This indicates that even the genes directly involved in AND, IND, and SKA metabolism have a small contribution for the trait phenotypic variance.

In our study, several genes associated with sexual development (TCF21, NME5, PTGFR, KCNQ1, UMODL1) were identified. TCF21 (*Transcription Factor 21*) is involved in testis growth and development in chicken [74]. In mice, TCF21 is also the first direct downstream target of the male *sex determining factor* (SRY) and both have similar effects on Sertoli cell differentiation and embryonic testis development in rats [75]. NME5 (*Nucleoside diphosphate kinase homolog 5*, also known as *NME/NM23 family member 5 gene*) is considered the best candidate for sperm concentration in Landrace [55]. According to Munier et al. [76], this gene is highly and specifically expressed in testis and the encoded protein is important for the initial stages of spermatogenesis. Choi et al. [77] reported that, when expression of NME5 is reduced, the spermatids in the testes become more sensitive to oxidative stress, leading to DNA damage and reduced sperm cell numbers, showing that this gene plays a crucial role in spermiogenesis. PTGFR (*Prostaglandin F Receptor*) is known to affect sexual behavior and to stimulate testicular hormone secretion in boars [78,79]. Epimutations (hypermethylation) in the KCNQ1 (*Potassium voltage-gated channel subfamily Q member 1*) gene have been reported in association with poor semen traits or male infertility [80,81].

One of the identified genes (UMODL1, *Uromodulin Like 1*) has been reported to be associated with female fertility, and, in particular, with oocyte fecundity. It is a gonadotropin-responsive gene expressed in both oocytes and the thymic medulla in mice. Mice carrying an extra allele of UMODL1 have drastically reduced litter sizes and increased premature infertility [82]. Reports suggest that disruptions to UMODL1 function may accelerate ovarian senescence [82]. As AND synthesis is stimulated along with other steroids, a relationship between BT and reproduction traits has been hypothesized. Genetic correlations between AND concentration and several female reproduction traits, including the total number of piglets born, have been reported in the literature [7].

Several candidate genes (CTSD, SYT8, TNNI2, CD81, EGR1, GIPC2, MIGA1, NEGR1, CCSER1, MTMR2, LPL, ERFE) related to adipocyte biogenesis, lipid metabolic processes and deposition, and to variations in fat thickness and intramuscular fat were also identified. CTSD (*Cathepsine D*) encodes for an aspartic lysosomal proteinase. Transcription of this gene is initiated from several sites, including one which is a start site for an estrogen-regulated transcript. The gene has been associated with growth, fat deposition, and production traits in the Italian Large White pig population [83,84]. It is worth mentioning that high cathepsin activity of porcine skeletal muscle is correlated to defects of dry-cured hams associated with excessive meat softness [85] and tainted hams have been associated to increased proteolysis and impaired organoleptic quality [86]. *Synaptotagmin 8* (SYT8) encodes a member of the synaptotagmin protein family, which includes membrane proteins involved in neurotransmission and hormone secretion. The Ca^2+^ non-binding isoform syt 8 has been detected in insulin-secreting cells. Glucose stimulated expression of SYT8 in human islets and SYT8 knock-down have shown to impair insulin release [87], even though the role of syt 8 in insulin secretion remains unclear [88].

TNNI2 (*Troponin I2*) gene has been associated with fat percentage, lean meat percentage, loin eye area, thorax–waist backfat thickness, and average backfat thickness in pigs [89]; TNNI2 gene affected significantly muscle pH, color, marbling, and intramuscular fat content [90]. CD81 (*Cluster of differentiation 81*) is a beige adipocyte progenitor cell marker and is required for de novo beige fat biogenesis; “beiging” of white adipose tissue is an adaptive process initiated as a response to cold temperatures in which numerous mitochondria-enriched thermogenic adipocytes with multi-locular lipid droplets (i.e., beige adipocytes) emerge within white adipose tissue [91]. EGR1 (*Early growth response 1*) influences adipocyte differentiation and was found to have an anti-adipogenic action [92], acting in opposition to EGR2. The regulation of both is required for maintaining appropriate levels of adipogenesis. *Neuronal growth regulator 1* (NEGR1), *Mitoguardin 1* (MIGA1, also known as protein FAM73A), and *GIPC PDZ domain containing family member 2* (GIPC2), are responsible for genetic predisposition to common forms of obesity in human, especially those resulting from deposition of subcutaneous fat, and they have been associated to subcutaneous fat thickness in a combined human and pig study [93]. *Coiled-Coil Serine Rich Protein 1* (CCSER1) was identified as a candidate gene for backfat thickness in Chuying-black pigs [94]. MTMR2 (*Myotubularin related protein 2*) was proposed as a functional candidate gene for intramuscular fat content in GWAS and signatures of selection studies in a Duroc pig population selected for intramuscular fat content [95]. *Lipoprotein lipase* (LPL) can hydrolyze the lipoprotein triacylglycerols from cycling and release free fatty acid for skeletal muscle uptake. The increased expression of the gene indicates that the ability to uptake exogenous fatty acid and synthesize triacylglycerols could be increased, which could intensify fat deposition [96]. ERFE (*Myonectin*, also known as *Erythroferrone*) is associated with iron metabolism, but is also a key player in regulating local and systemic lipid metabolism [97]. In particular, when fed a high-fat diet, myonectin-knockout mice had significantly elevated very-low-density-lipoprotein–triglyceride and impaired lipid clearance from circulation following an oral lipid load. Fat distribution between adipose tissue and liver was also altered. Greater fat storage in adipocytes was associated with increased postprandial lipoprotein lipase activity in adipose tissue [97].

The large number of candidate genes related to fat metabolism can be ascribed to the well-known relationship between sexual steroid levels and fat deposition. According to Kelly and Jones [35], reduced tissue testosterone facilitates triglyceride storage in adipocytes by allowing increased lipoprotein lipase activity and stimulating pluripotent stem cells to mature into adipocytes. In addition, increased adipocyte mass is associated with greater insulin resistance and further inhibits testosterone synthesis [38]. Due to the relationship between BT compound concentrations and fat deposition, the pig line investigated, which is selected for ham quality and not for lean growth, might have influenced the results obtained and might explain the discrepancies in the identified genes between our study and the literature. In a recent study performed on the same pig line, significant genetic correlations were detected between BT compound concentrations measured in purebred pigs and carcass backfat or ham subcutaneous fat measured in crossbred heavy pigs [98].

**Table 4:** Candidate genes identified for androstenone (AND), indole (IND), and skatole (SKA), considering the genomic regions associated; *Sus scrofa* chromosome (SSC), position of the gene, and gene name and functions are reported.

Trait	SSC	Position (Mb)	Gene	Function	References
IND, SKA	1	29.98–29.98	TCF21	Associated with testis growth and development	[74]
AND	2	1.19–1.20	CTSD	Encodes lysosomal aspartyl proteinase	[83]
AND	2	1.25–1.25	SYT8	Encodes a member of the synaptotagmin protein family (neurotransmission and hormone secretion)	[87,88]
AND	2	1.25–1.25	TNNI2	Associated with intramuscular fat content	[89,90]
AND	2	1.63–1.64	CD81	Associated with adipocyte biogenesis	[91]
AND	2	1.68–1.99	KCNQ1	Associated with male infertility	[80]
IND	2	140.13–140.17	NME5	Associated with spermiogenesis	[76]
IND	2	140.44–140.45	EGR1	Associated with adipocyte differentiation	[92]
IND	6	134.73–134.77	PTGFR	Binds PGF2α, associated with prostaglandin F receptor, affects sexual behavior, stimulates testicular hormone secretion	[78,79]
AND, IND	6	135.08–135.22	GIPC2	Associated with subcutaneous fat thickness	[93]
AND, IND	6	135.42–135.51	MIGA1	Associated with backfat thickness	[93]
AND, IND	6	140.78–141.65	NEGR1	Associated with subcutaneous fat thickness	[93]
AND	8	127.73–128.95	CCSER1	Associated with backfat thickness and loin depth	[94]
AND	9	27.85–27.97	MTMR2	Associated with lipid metabolic processes	[95]
IND	14	4.11–4.14	LPL	Hydrolyses triacylglycerols in plasma lipoproteins	[96]
SKA	13	204.84–204.87	MX1	Associated with boar taint	[37]
SKA	13	205.43–205.49	UMODL1	Involved in the regulation of ovarian follicle development	[82]
SKA	15	137.73–137.74	ERFE	Promotes lipid uptake into adipocytes and hepatocytes	[97]

## 4. Conclusions

In the current study, the accuracy in predicting EBV using only pedigree data or only genomic data was compared. For androstenone, the exploitation of genomic data led to significantly more accurate EBV predictions compared to those obtained using pedigree data, whereas for IND and SKA, the accuracies were not significantly different. In general, genomic estimated breeding values can be used in selective breeding aimed to decrease boar taint compounds in entire male pigs, avoiding the challenging and expensive routine BT compound phenotyping required by traditional pedigree-based selection. Hence, genomic selection represents a promising alternative to male piglet castration and allows pigs to be slaughtered at the age and body weight required by product specifications of protected designation of origin dry-cured hams. Genome-wide association studies revealed that boar taint compounds are influenced by a large number of loci, each explaining a small percentage of the variance. The polygenic nature of boar taint makes genomic selection strategies particularly beneficial as genomic BLUP ignores any information about the genetic architecture of a trait and assumes that each marker equally contributes to the genetic variance. Post-GWAS analyses of biological processes led to the identification of 18 candidate genes encoding receptors and enzymes associated with BT (MX1), reproduction traits (TCF21, NME5, PTGFR, KCNQ1, UMODL1), and fat metabolism (CTSD, SYT8, TNNI2, CD81, EGR1, GIPC2, MIGA1, NEGR1, CCSER1, MTMR2, LPL, ERFE). The large number of candidate genes (12 out of 18) related to fat metabolism might be explained by the well-known relationship between sexual steroid levels and fat deposition and be, in part, ascribed to the pig line investigated, which is selected for ham quality and not for lean growth.

## Figures and Tables

**Table 1:** Number of animals with phenotypic data, mean (±SD) concentration of boar taint compounds before and after log-transformation, and heritability (h^2^ ± SE) of the investigated traits.

Trait	N	Mean (± SD) before Log-Transformation	Mean (± SD) after Log-Transformation	h^2^
Androstenone (ng/g)	1051	1162.8 ± 1018.2	6.77 ± 0.76	0.30 ± 0.08
Indole (ng/g)	1073	21.7 ± 25.0	2.63 ± 0.93	0.43 ± 0.09
Skatole (ng/g)	1069	48.3 ± 70.6	3.31 ± 1.04	0.51 ± 0.09

**Table 2:** Pearson correlations between predicted EBV and adjusted phenotypes ($r_{EBV,\;y_{adj}}$), corresponding accuracies ($R_{EBV,\;BV}$) and β coefficients.

Trait	${r_{EBV,\;y_{adj\;}}}^{1}$		${R_{EBV,\;BV\;}}^{2}$		β	
	PBLUP	GBLUP	PBLUP	GBLUP	PBLUP	GBLUP
Androstenone	0.198	0.319 **	0.362	0.582 **	0.893	0.887
Indole	0.292	0.273	0.446	0.417	1.008	0.663
Skatole	0.326	0.354	0.452	0.491	0.963	0.768

^1^ Pearson correlation between predicted EBV and adjusted phenotypes (**y_adj_**). ^2^ Accuracy computed as in Equation (2); ** indicates statistically significant differences (*p* < 0.01) between PBLUP and GBLUP.

**Table 3:** QTL regions identified for androstenone, indole, and skatole. *Sus scrofa* chromosome (SSC), position of QTL region, number of SNPs within the QTL region and percentage of variance explained by the windows are reported.

Trait	SSC	QTL Region (Mb)	N SNP	Variance Explained (%)	Other Associated Boar Taint Compound
Androstenone	1	15.05–15.44	14	0.56	
	2	1.23–1.54	13	0.58	
	2	156.30–156.65	12	0.75	
	6	135.01–135.40	15	0.90	Indole
	6	140.66–141.06	12	0.53	Indole
	7	18.15–18.54	11	0.62	
	8	128.63–128.99	16	0.58	
	9	27.88–28.27	12	0.73	
Indole	1	29.76–30.15	9	0.52	Skatole
	2	140.25–140.64	16	0.56	
	4	15.44–15.74	8	0.58	Skatole
	4	141.08–141.46	9	0.86	
	6	134.85–135.24	15	0.78	Androstenone
	6	140.95–141.31	14	0.63	Androstenone
	8	17.93–18.32	11	0.90	
	14	3.77–4.13	14	0.67	
Skatole	1	30.00–30.38	13	0.58	Indole
	4	15.44–15.74	8	0.50	Indole
	13	9.55–9.94	23	0.54	
	13	204.99–205.38	16	0.89	
	15	137.53–137.93	10	0.64	
	18	0.76–1.12	9	0.61	

## Data Availability

Restrictions apply to the availability of these data. Data were obtained from Gorzagri (Fonzaso, Italy) and are available from the authors with the permission of Gorzagri.
